# Metformin Improves Diabetic Bone Health by Re-Balancing Catabolism and Nitrogen Disposal

**DOI:** 10.1371/journal.pone.0146152

**Published:** 2015-12-30

**Authors:** Xiyan Li, Yuqi Guo, Wenbo Yan, Michael P. Snyder, Xin Li

**Affiliations:** 1 Department of Genetics, Stanford University, Stanford, CA 94305–5120, United States of America; 2 Basic Science and Craniofacial Biology, New York University College of Dentistry, New York, NY 10010, United States of America; 3 Department of Biology and Chemistry, Nyack College, New York, NY 10013, United States of America; Baylor College of Medicine, UNITED STATES

## Abstract

**Objective:**

Metformin, a leading drug used to treat diabetic patients, is reported to benefit bone homeostasis under hyperglycemia in animal models. However, both the molecular targets and the biological pathways affected by metformin in bone are not well identified or characterized. The objective of this study is to investigate the bioengergeric pathways affected by metformin in bone marrow cells of mice.

**Materials and Methods:**

Metabolite levels were examined in bone marrow samples extracted from metformin or PBS -treated healthy (Wild type) and hyperglycemic (diabetic) mice using liquid chromatography-mass spectrometry (LC-MS)-based metabolomics. We applied an untargeted high performance LC-MS approach which combined multimode chromatography (ion exchange, reversed phase and hydrophilic interaction (HILIC)) and Orbitrap-based ultra-high accuracy mass spectrometry to achieve a wide coverage. A multivariate clustering was applied to reveal the global trends and major metabolite players.

**Results:**

A total of 346 unique metabolites were identified, and they are grouped into distinctive clusters that reflected general and diabetes-specific responses to metformin. As evidenced by changes in the TCA and urea cycles, increased catabolism and nitrogen waste that are commonly associated with diabetes were rebalanced upon treatment with metformin. In particular, we found glutamate and succinate whose levels were drastically elevated in diabetic animals were brought back to normal levels by metformin. These two metabolites were further validated as the major targets of metformin in bone marrow stromal cells.

**Conclusion:**

Overall using limited sample size, our study revealed the metabolic pathways modulated by metformin in bones which have broad implication in our understanding of bone remodeling under hyperglycemia and in finding therapeutic interventions in mammals.

## Introduction

Diabetes mellitus (DM) is a group of chronic diseases that are characterized by high blood glucose levels and are becoming a global epidemic [1]. If not controlled, DM adversely affects many tissues including bone [2], a dynamic organ that undergoes continuous remodeling to maintain its quantity and quality. Patients with diabetes are at greater risk of fractures mostly due to not only extra-skeletal factors, such as propensity to fall, but also bone quality alteration, which reduces bone strength. The balance between bone resorption by osteoclasts and bone formation by osteoblasts is critical for skeletal homeostasis [3–5]. Type-1 and Type-2 diabetes are associated with an increased risk of osteoporosis and fragility fractures [2] and osteoclastogenesis is usually enhanced and leading to accelerated bone resorption in DM [6–8].

It has been recognized that metformin, an oral anti-diabetic medicine and an insulin sensitizer, improves bone metabolism and turnover. Metformin-treated T2D patients have decreased risks of bone fracture [9]. The osteogenic effects of metformin have been documented in cellular and rodent models: 1), metformin promotes osteoblast differentiation and inhibits adipocyte differentiation in cultured rat marrow mesenchymal stem cells, likely through inhibition of PPARγ, a nuclear receptor that regulates lipid and glucose metabolism [10]; 2), metformin increases trabecular bone formation through activation of AMPK signaling in osteoblastic cells, a major intracellular pathway that senses energy starvation [11]. Conversely, metformin directly inhibits bilateral ovariectomy-induced bone loss in rats [12] and osteoclastogenesis[13], which is also evidenced by a significant reduction of serum bone resorption marker (-12.7%) in male diabetic patients taking metformin [14]. Interestingly, the osteogenic effects by metformin may be specific to diabetes as bone formation is not promoted by metformin in mice with normal blood glucose levels [15]. These observations suggest that metformin may elicit a complex mode of actions in bone maintenance, which are associated with metabolic conditions, although the causality is still unclear.

Several observations point to the role of metformin as a metabolic switch that simultaneously inhibits anabolism and activates catabolism [16–18], including: 1) AMPK activation that stimulates β-oxidation of fatty acids and ketogenesis and inhibits cholesterol and lipid synthesis; 2) inhibition of respiratory complex I that reduces both NADH consumption and the generation of endogenous ROS (1–3% of total electron transport from oxidative phosphorylation); 3) suppression of cAMP elevation that inhibits lipogenesis. These intracellular consequences of metformin administration directly counteract the effects of insulin, and may thus alleviate harmful effects resulting from elevated insulin levels in T2D. However, aside from the aforementioned observations and correction of hyperglycemia, it remains largely unclear how metformin modulates its molecular targets in a cell. Since hyperglycemia is a chronic metabolic disease, metabolic deterioration in diabetic bone marrow could be the direct and fundamental reason for compromised bone quality and quantity. It would be intriguing to investigate the metabolomic responses that mediate the osteogenic effects of metformin to reduce skeletal fracture risk under T2D conditions. To date, there have been no studies to examine the skeletal metabolic effects by metformin in diabetes.

In this study, we investigated the mechanism of metformin actions in bone maintenance in hyperglycemic (MKR, Stands for MCK-KR-hIGF-IR) and wild type mice. MKR mice rapidly develop severe diabetes due to a dominant negative mutant of human IGFI receptor (hIGF-IR) that is specifically expressed in skeletal muscle by muscle-creatine kinase MCK [19]. We applied an untargeted high performance liquid chromatography-mass spectrometry (LC-MS) approach which combined multimode chromatography (ion exchange, reversed phase and hydrophilic interaction (HILIC)) and Orbitrap-based ultra-high resolution mass spectrometry to achieve a wide coverage of cellular metabolites. A multivariate clustering was applied to reveal the global metabolic interactions between metformin and diabetes and pinpoint major metabolite players. The functions of these metabolites supported that metformin might target these metabolites to regulate bone homeostasis under diabetic condition.

## Materials and Methods

### Animals

All animal experiments were carried on with the compliance of New York University Institutional Animal Care and Use Committee (IACUC). The protocol approval number was 111004. Hyperglycemic mouse model MKR breeders were generously provided by LeRoith and colleagues [19]. By creating a dominant negative mutant of human IGFI receptor, MKR mouse rapidly develops severe diabetes due to a decrease in glucose uptake and insulin resistance [19]. Friend Virus B (FVB) background wildtype (WT) breeders were ordered from Jackson Lab (Bar Harbor, ME).

### Bone mineral density (BMD) and bone formation

Twelve-week-old male WT and MKR mice were each randomly assigned to two groups (n = 4 in each group) and received daily intraperitoneal (I.P) injections of vehicle (PBS) (HyClone, Logan, UT) or metformin (Met, 200mg/kg BW) (Calbiochem, San Diego, CA) in 50ul volume for 14-days. After euthanization, long bones from hind limbs were dissected. Paraformaldehyde-fixed femora were evaluated with μCT using a SkyScan 1172 high-resolution scanner (Bruker microCT, Kontich, Antwerp, Belgium) with 60kV votage and 167μA current. Reconstruct cross section with 9.7 μm pixel size and scan images analyzed by using CTan (V.1.14.4) to generate the Regions of interest. CTVox was used to generate the 3D image with a 60 degree view angle and (0, 0, 4) camera position afterwards.

### Metabolomics sample preparation

Total bone marrow cells were obtained by cutting both ends of long bones open and flushing out with PBS into Eppendorf Protein LoBind tubes (1.5ml). After washing twice with PBS, cells were counted the using a hemocytometer. Then 10 million of the cells were frozen immediately and referred as frozen bone marrow cells. At the time of extraction for HPLC, frozen cells were mixed with dry ice-cool 80% methanol (mass-spec grade) (Fisher Scientific, Hampton, NH) at a ratio of 30 μL/million cells, and then quickly thawed on heat block set at 50°C for 5 min. The suspension was then processed by three rounds of 1 min vortex at max speed, chilled briefly on dry ice. The mixture was incubated at 4°C for 1 hour before centrifuge at 20,000 x g for 20 min at 4°C. The supernatant was stored at -20°C and used as metabolite extract for LC-MS analysis. For LC-MS analysis, the metabolite extract was transferred to 150 μL deactivated glass insert housed in Waters 2-ml brown MS vials (Waters Corporation, Milford, MA). Chemical standard solution was prepared from synthetic complete mixture from Sigma-Aldrich (Y1501) (St. Louis, MO, USA) at a concentration of 19 μg/ml 80% methanol (mass-spec grade).

### LC-MS Acquisition

Metabolite extract was analyzed in a platform that consists of Waters UPLC-coupled Exactive Orbitrap Mass Spectrometer (Thermo Scientific, Waltham, MA), using a mix-mode OPD2 HP-4B column (4.6x50 mm) (Shodex, Showa Denko, Tokyo, Japan). The column temperature was maintained at 45°C. Five microliters of each sample maintained at 4°C was loaded by the autosampler (Fisher Scientific, Hampton, NH) in partial loop mode for three times at positive mode and negative mode, respectively. The binary mobile phase solvents were: A, 10 mM NH_4_OAc in 10:90 Acetonitrile:water; B, 10 mM NH_4_OAc in 90:10 Acetonitrile:water. Both solvents were modified with 10 mM HOAc for positive mode acquisition, or 10 mM NH_4_OH for negative mode. The 30-min gradient for both modes was set as: flow rate, 0.1 ml/min; 0–15 min, 99% A, 15–18 min, 99% to 1% A; 18–24 min, 1% A; 24–25 min, 1% to 99% A; 25–30min, 99% A. The MS acquisition was in profile mode and performed with an ESI probe, operating with capillary temperature at 275°C, sheath gas at 40 units, spray voltage at 3.5 kV for positive mode and 3.1 kV for negative mode, Capillary voltage at 30 V, tube lens voltage at 120 V, and Skimmer voltage at 20 V. The mass scanning used 100,000 mass resolution, high dynamic range for AGC Target, 500 milliseconds as Maximum Inject Time, and 75–1200 m/z as the scan range. The system was operated by Thermo Xcalibur v2.1 software (Thermo Scientific, Waltham, MA). All chemicals were from Sigma-Aldrich (St. Louis, MO) if not specifically mentioned.

### LC-MS data analysis

The raw data files generated from LC-MS were centroided with PAVA program [20] and converted to mzXML format by an in-house R script (distribution upon request). Mass feature extraction was performed with XCMS v1.30.3 [21]. The mass features were then manually searched against the Metlin metabolite database using 5 ppm mass accuracy. Retention time matching with compounds in the standard mixture was also performed for a portion of the metabolite hits. Only those mass features with a KEGG metabolite entry were retained for further analysis. The scored mass features were then clustered with SIMCA v13.03 (Umetric). The clustering used the O2PLS-DA model and unit-variance scaling. The KEGG IDs of those metabolites with high discriminatory power for metformin treatment in hyperglycemic mice were used for pathway enrichment analysis with IMPaLA [22].

### Focus network construction

The KEGG pathway enrichment listed in Table 1 were imported and merged by matching their KEGGIDs in Cytoscape (v3.2.0). Analysis of network connectivity by the number of edges in this focus network was calculated, and the node sizes and labels were formatted according to the number of edges. The focus network is presented in organic layout format in Cytoscape.

**Table 1:** Pathway enrichment analysis of metformin-suppressed metabolites from MKR mice.

KEGG pathway_name	Pathway ID	Overlapping Metabolites	All Metabolites	P_metabolites_	Q_metabolites_
Alanine, aspartate and glutamate metabolism	KO00250	C00158;C01042;C00022;C00025;C00026;C00232;C00042;C00122	28	7.67E-09	1.39E-06
Citrate cycle (TCA cycle)	KO00020	C00158;C00149;C00417;C00026;C00042;C00022;C00122	20	1.44E-08	2.28E-06
Glyoxylate and dicarboxylate metabolism	KO00630	C00158;C00160;C00149;C00417;C00025;C00026;C00042;C00898;C00022	58	2.60E-07	2.40E-05
Butanoate metabolism	KO00650	C01089;C00022;C00025;C00026;C00232;C00042;C00122	41	3.18E-06	0.000193
Histidine metabolism	KO00630	C00860;C02835;C00025;C00026;C00785;C05570;C00439	45	6.11E-06	0.000333
Vitamin B6 metabolism	KO00750	C00022;C00118;C00026;C00279;C00847;C00232	32	9.62E-06	0.000457
Pentose phosphate pathway	KO00030	C00117;C00022;C00118;C00279;C00221;C00121	35	1.66E-05	0.000735
Glutathione metabolism	KO00480	C01672;C00072;C00750;C00025;C00051;C00134	38	2.71E-05	0.00111
Cysteine and methionine metabolism	KO00270	C00022;C00094;C00979;C00059;C00491;C03145;C00051	57	3.05E-05	0.00124
Arginine and proline metabolism	KO00330	C00750;C00025;C00437;C04281;C00581;C00022;C00134;C00122	90	8.63E-05	0.00273
Ascorbate and aldarate metabolism	KO00053	C00072;C00800;C00022;C00191;C00026;C01620	47	9.41E-05	0.00295
Taurine and hypotaurine metabolism	KO00430	C00022;C00025;C00026;C00094	22	0.000377	0.00731

The KEGG IDs for all 65 metabolites with a high pq2 score (>0.05, see Fig 2B) were used for the pathway enrichment analysis (http://impala.molgen.mpg.de/impala/impala). The Q-value cut-off were < 0.01.

### Statistic Analysis

Following a Guideline to Univariate Statistical Analysis for LC/MS-Based Untargeted Metabolomics-Derived Data [23], we performed multivariate analysis and univariate analysis to capture the overall metabolomic patterns between healthy and diabetic groups. We used multivariate analysis of manually curated high-confidence mass features. We used univariate t-test to score the potential significance. Detailed statistic analysis is included in the legend of each figure. For the rest, analysis of variance (ANOVA) was used when study subjects were more than 2 groups, followed by the Bonferroni t-test. Two-tailed student’s t-test was used to compare the difference between two experiment groups. A value of P < 0.05 was considered to statistically significant.

## Results

### Metformin specifically alleviated the BMD reduction in MKR mice

**Fig 1:**
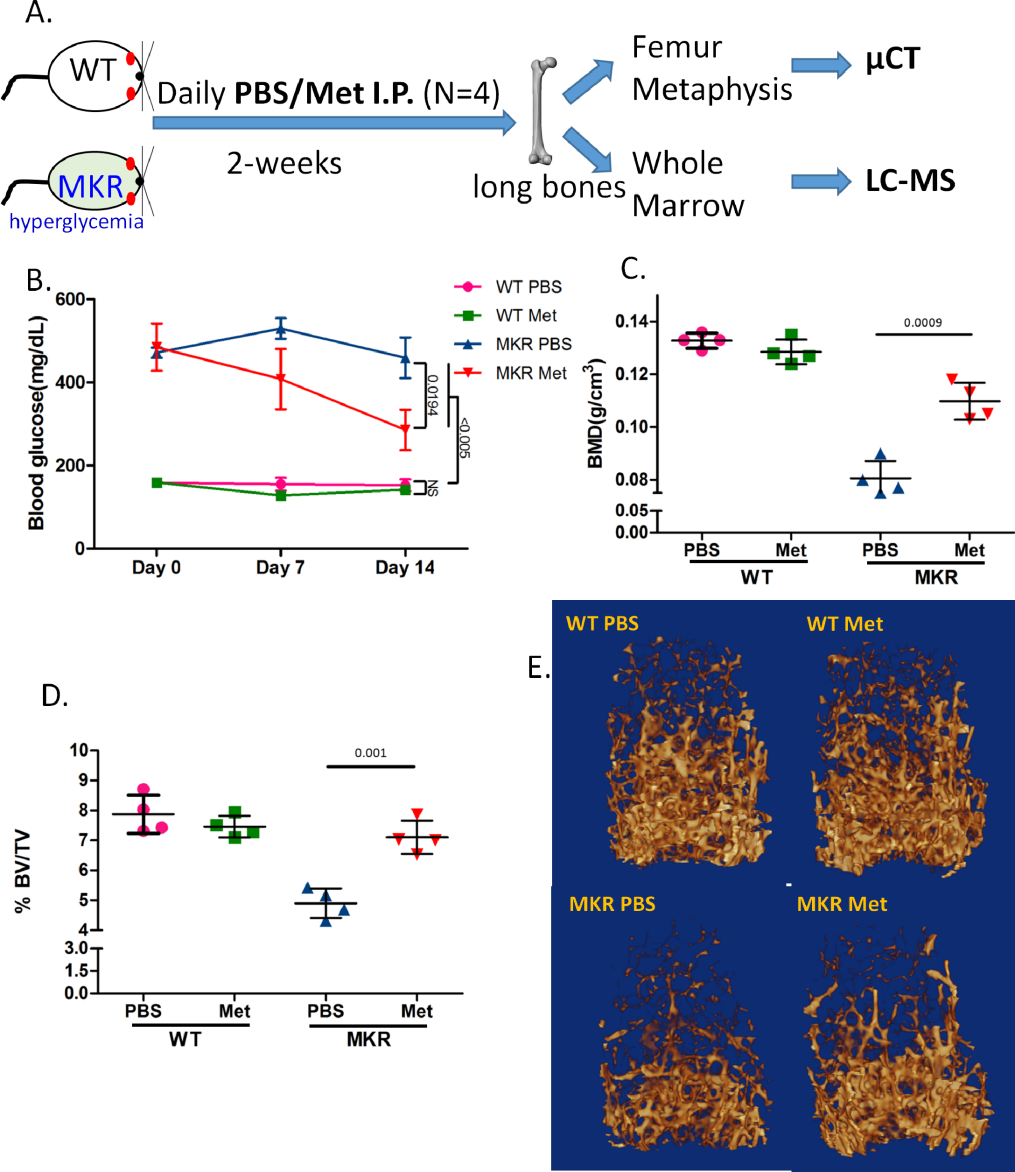
Metformin protected bone loss in hyperglycemic mice. A. Scheme of the experimental design in which 12-week-old male wild type (WT) and hyperglycemic (MKR) mice were treated with vehicle (PBS) or metformin intraperitoneally daily for 2-weeks. Long bones from both side hind limbs were isolated at the end of experiments from euthanized mice. One femur was processed for microCT (μCT). Whole marrow flushed from the other femur and two tibiae were immediately frozen until LC-MS assay. B. Blood glucose levels (mean ±SEM, n = 4) measured weekly over the 2-week treatment. C. Bone mineral density (BMD, n = 4) of distal femoral metaphyseal regions. D. Bone volume versus tissue volume (BV/TV, n = 4) of distal femoral metaphyseal regions. E. Representative images of μCT reconstruction.

MKR mice have slender bones and exhibit skeletal fragility and susceptibility to fracture due to reduced transverse bone accrual and increased osteoclastogenesis [24]. Our study (depict in Fig 1A) confirmed the hyperglycemia and compromised basal bone condition in MKR mice in comparison to WT (Fig 1B and 1C). Interestingly, metformin treatment significantly and specifically improved the bone quality: both the trabecular bone mineral density (BMD) (Fig 1C) and the amount of bone volume (BV/TV) of the femoral distal metaphyseal regions (Fig 1D) were elevated by metformin in MKR mice, but not in WT mice, as demonstrated by μCT 3D analysis (Fig 1E).

### Metformin differentially shifts the overall bone marrow metabolite profiles between WT and MKR mice

**Fig 2:**
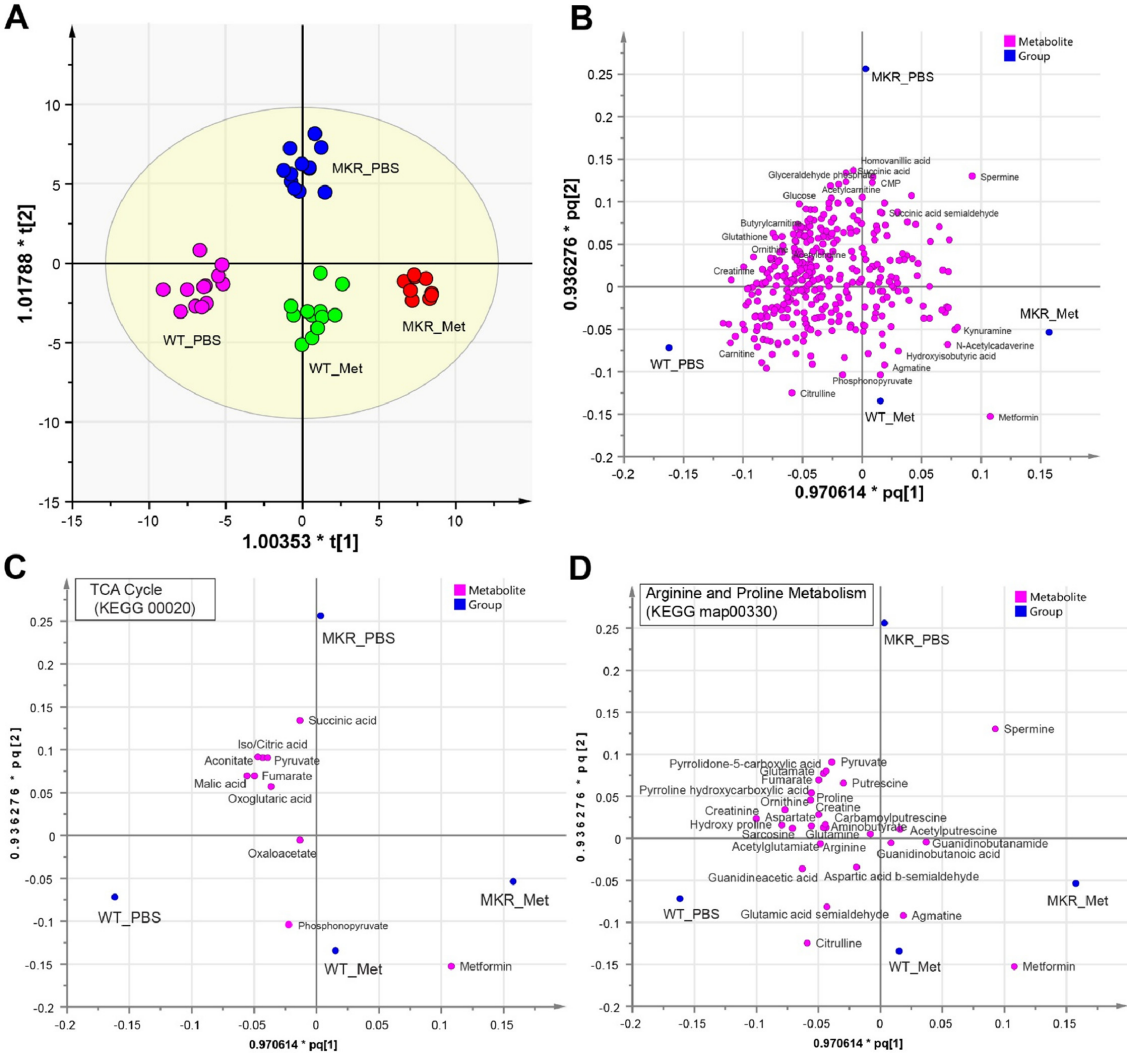
Metformin shifted metabolomics profiles of bone marrow in both wild-type and hyperglycemic mice. A. The clustering scatter plot of 345 metabolites from bone marrow cells of WT and MKR mice upon metformin (Met) or control (PBS) treatment. Each dot represents one of 3 technical replicates from each of 4 biological samples (for MKR_Met, n = 3). The clustering used the O2PLS-DA model and unit-variance scaling in SIMCA. R2Y = 0.942. Q2 = 0.591. B. The scatter plot of metabolite contribution to clustering in panel A. The first and second predictive components (R2 = 0.631) from the O2PLS-DA model in panel A. are superimposed with their p and q plots. Each magenta dot represents a metabolite. Each blue dot represents the reference point for each sample group. The names were showed for a few metabolites with high discriminatory power between sample groups. Plots were generated in SIMCA. C-D. The scatter plot of metabolite contribution in two representative KEGG metabolism pathways (as in Fig 2B). All metabolites in the displayed pathway are showed if detected in this study regardless of their statistical significance. Metformin is always included as reference.

To examine the metabolic change, we performed metabolomics profiling with LC-MS (see Methods for instrument settings and analysis procedure) using multimode chromatography (HILIC). We extracted 14062 mass features (each defined by a pair of retention time and accurate mass) from positive mode and 5959 mass features from negative mode. A total of 346 unique metabolites (229 from positive mode, 130 from negative mode) were identified after manual validation with database search and comparing with compound standards, and used in multivariate clustering analysis. As the clustering results showed (Fig 2A), metformin clearly altered the metabolite profiles of mouse bone marrow, as each sample groups resided tightly in discrete clusters with high statistical confidence (R2Y = 0.942. Q2 = 0.591). The metabolomic changes induced by metformin exhibited similar horizontal shift (primary change) for both WT and MKR, suggesting metformin exerts similar global effects on metabolism in WT and MKR mice. However, metformin elicited additional metabolomic changes only in MKR mice, as demonstrated by the vertical shift (secondary) in the clustering plot (Fig 2B). These additional changes may explain the benefits in bone health only observed in MKR mice but not in WT mice when treated with metformin.

### Metformin preferentially affects energy and nitrogen metabolic pathways

Based on the model defined by clustering in Fig 2A, the contribution of each individual metabolite was assessed for their discriminatory power, which can be approximated by their distance to each group reference placeholder (Fig 2B). As the benchmark for this clustering, metformin resided unequivocally in close proximity to both WT and MKR groups with metformin treatment (Fig 2B), supporting the validity of this analysis. Since the metabolites that resided in the upper section (quadrants I and II) in Fig 2B represent those with high discriminatory power specific to MKR only, we used arbitrary criteria (Y-variable > 0.05) to select 65 metabolites as high confidence variables for pathway enrichment analysis (see Methods). Given that a majority of these metabolites are suppressed by metformin (judged by their closer distance to MKR_PBS than to MKR_Met), these results suggest that metformin acts by suppressing key metabolic pathways, and preferentially targets and suppresses energy metabolism (e.g. TCA cycle) and nitrogen metabolism (e.g. urea cycle and several other amino acid pathways) (Table 1). Metformin also exacerbated the metabolomic suppression that already exists in MKR, as more metabolic pathways were suppressed by metformin in addition to those already suppressed in untreated MKR (Table 2). Pathway-wise contribution of individual metabolites also showed that both TCA cycle and arginine/proline metabolism (hosting urea cycle) were shifted away from MKR control to MKR with metformin and to WT groups (Fig 2C and 2D). These observations provide metabolomic evidence to support several previous reports, including 1) depletion of TCA cycle by metformin in rat liver and breast cancer stem cells [25, 26], 2) increase of aerobic glycolysis and reduction of glucose metabolism by metformin through the TCA cycle in breast cancer cells [27], and 3) indirect regulation of urea cycle through metformin-mediated AMPK activation [28].

**Table 2:** Pathway enrichment analysis of metformin-suppressed metabolites from MKR mice.

KEGG pathway name	Overlapping metabolites	All metabolites	P_metabolites_	Q_metabolites_
**Down regulated in MKR (N = 82)**				
Glycine, serine and threonine metabolism	11	50	2.10E-09	6.57E-08
Arginine and proline metabolism	11	91	1.37E-06	2.32E-05
Purine metabolism	10	92	1.14E-05	0.000178
Cysteine and methionine metabolism	7	57	0.000118	0.00152
Alanine, aspartate and glutamate metabolism	5	28	0.000196	0.00226
Taurine and hypotaurine metabolism	4	22	0.000832	0.00736
Nicotinate and nicotinamide metabolism	5	49	0.00273	0.0186
**Down regulated by metformin (N = 125)**				
*Alanine*, *aspartate and glutamate metabolism*	13	28	2.74E-13	1.60E-11
Glyoxylate and dicarboxylate metabolism	14	58	9.65E-10	2.17E-08
*Arginine and proline metabolism*	17	91	1.01E-09	2.22E-08
*Glycine*, *serine and threonine metabolism*	13	50	1.47E-09	3.10E-08
Histidine metabolism	11	45	5.85E-08	1.08E-06
*Cysteine and methionine metabolism*	11	57	7.79E-07	1.08E-05
Citrate cycle (TCA cycle)	7	20	1.17E-06	1.52E-05
*Taurine and hypotaurine metabolism*	7	22	2.44E-06	3.06E-05
Pentose phosphate pathway	8	35	7.13E-06	7.58E-05
*Nicotinate and nicotinamide metabolism*	9	49	1.27E-05	0.000116
*Glutathione metabolism*	8	38	1.37E-05	0.000125
Butanoate metabolism	8	41	2.48E-05	0.000215
Oxidative phosphorylation	5	16	8.18E-05	0.000632
Glycerophospholipid metabolism	8	52	0.000147	0.00105
Sulfur metabolism	6	29	0.000192	0.00133
Choline metabolism in cancer	4	11	0.000232	0.0015
D-Glutamine and D-glutamate metabolism	4	12	0.00034	0.00214
*Purine metabolism*	10	92	0.000426	0.0026
Valine, leucine and isoleucine biosynthesis	5	23	0.000531	0.00315
Pyrimidine metabolism	8	66	0.000786	0.00449
Thiamine metabolism	5	30	0.00189	0.00987

The KEGG IDs of metabolites that lie >0.05 unit away from y = -x in Fig 2B were used as high-confidence metabolites specific to MKR for pathway enrichment analyses with IMPaLA. Similarly, the KEGG IDS of metabolites that lie > 0.05 unit away from y = x in Fig 2B were scored as high-confidence metabolites specific to metformin treatment in MKR for IMPaLA analyses. The Q-value cut-off were < 0.01 (except for “Nicotinate and nicotinamide metabolism” that is included for comparison purpose). No enrichment was found for both upregulated metabolite groups in MKR (N = 42) and in metformin treatment (N = 17). The pathways that found in both groups are in italics for the metformin-treatment group.

### Diverse response patterns of metabolites suggest complex consequences of metformin administration

**Fig 3:**
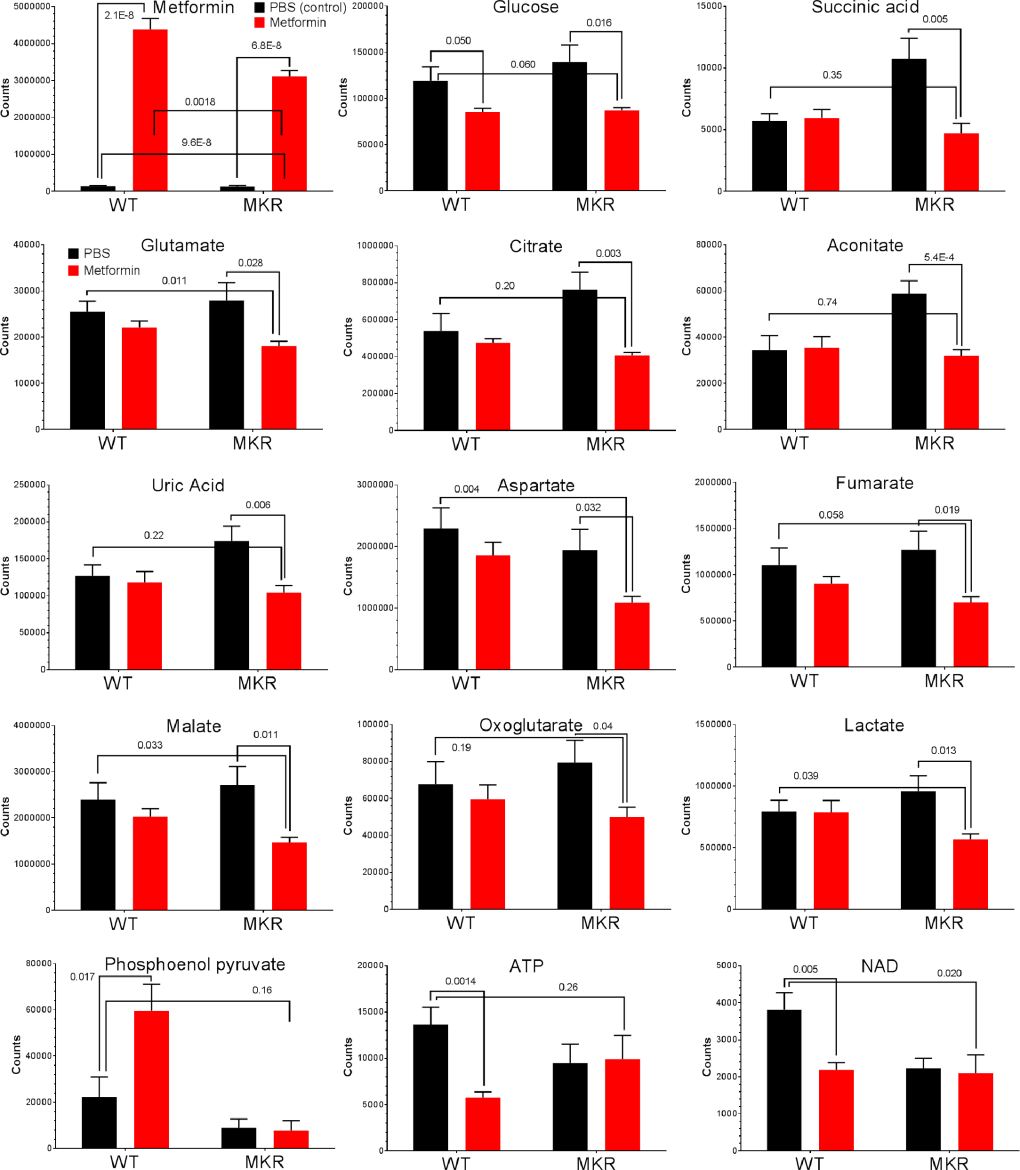
Individual metabolite levels in bone marrow affected by metformin. Each plot shows the mean ± SEM values of respective sample groups for one particular metabolite (n = 4 for WT_PBS, WT_Met, MKR_PBS, n = 3 for MKR_Met, each sample with technical triplicates). The p-values for t-test (two-tailed, unequal variance) is also showed whenever significant (< 0.05). Plots were generated in GraphPad Prism 6.

Our study also revealed a great diversity in how individual metabolites are affected by metformin (Fig 3). Interestingly, the metformin levels in MKR cells were only 71% of those in WT, suggesting the drug uptake/delivery in MKR may not be as effective as in WT. A number of metabolites thatincreased in MKR condition were restored to the levels in WT by metformin. Metabolites in this group include succinate, aconitate, uric acid and citrate which belong to the TCA cycle and glycolysis. These results indicate that metformin is resetting metabolic pathways such as TCA cycle and glycolysis in MKR bone marrows, which could be beneficial in protection against bone damage caused by diabetic conditions.

In contrast, metformin made no substantial differences in intracellular glucose concentration between WT and MKR, as in both cases metformin suppressed intracellular glucose significantly when compared to vehicle treatment. This intracellular glucose-lowering effect by metformin may trigger starvation response that in turn leads to increased glucose uptake through facilitated translocation of glucose transporters [29].

Incremental suppression was observed for metabolites like glutamate, aspartate, fumarate, oxoglutarate, malate and lactate, whose levels were normal in WT but lowered by metformin in MKR (Fig 3). These patterns suggest that the homeostatic control of these metabolites was defective in MKR mice, which was exacerbated by metformin and may arise as a consequence of the reestablished metabolic balance that is overall attenuated by metformin (e.g. ATP, see below). Phosphoenol pyruvate (PEP), in contrast, was elevated by metformin in WT but unaffected in MKR. This pattern suggests a hyper-active glycolysis pathway and corroborates increased glucose uptake by metformin [29].

Surprisingly, ATP and NAD (nicotinamide adenine dinucleotide), two metabolite benchmarks for cellular energy regeneration capacity, were greatly suppressed by metformin only in WT (Fig 3). In contrast, metformin failed to further suppress the already reduced ATP and NAD levels in MKR. These patterns indicated that the capacity of overall energy output in MKR was not further reduced by metformin, despite the observation that many related metabolites are affected. Instead, our results strongly suggest that metformin alleviates the hyperglycemic condition by modulating metabolic processes more than just energy generation.

The diversity in response patterns echoes the unexpected discovery and re-discovery of the roles metformin in medicine [2, 30–32], and might explain its elusive modes of action that is still at large after its first application in humans 70 years ago.

### Network analysis pinpoints the metabolic process targets of metformin

**Fig 4:**
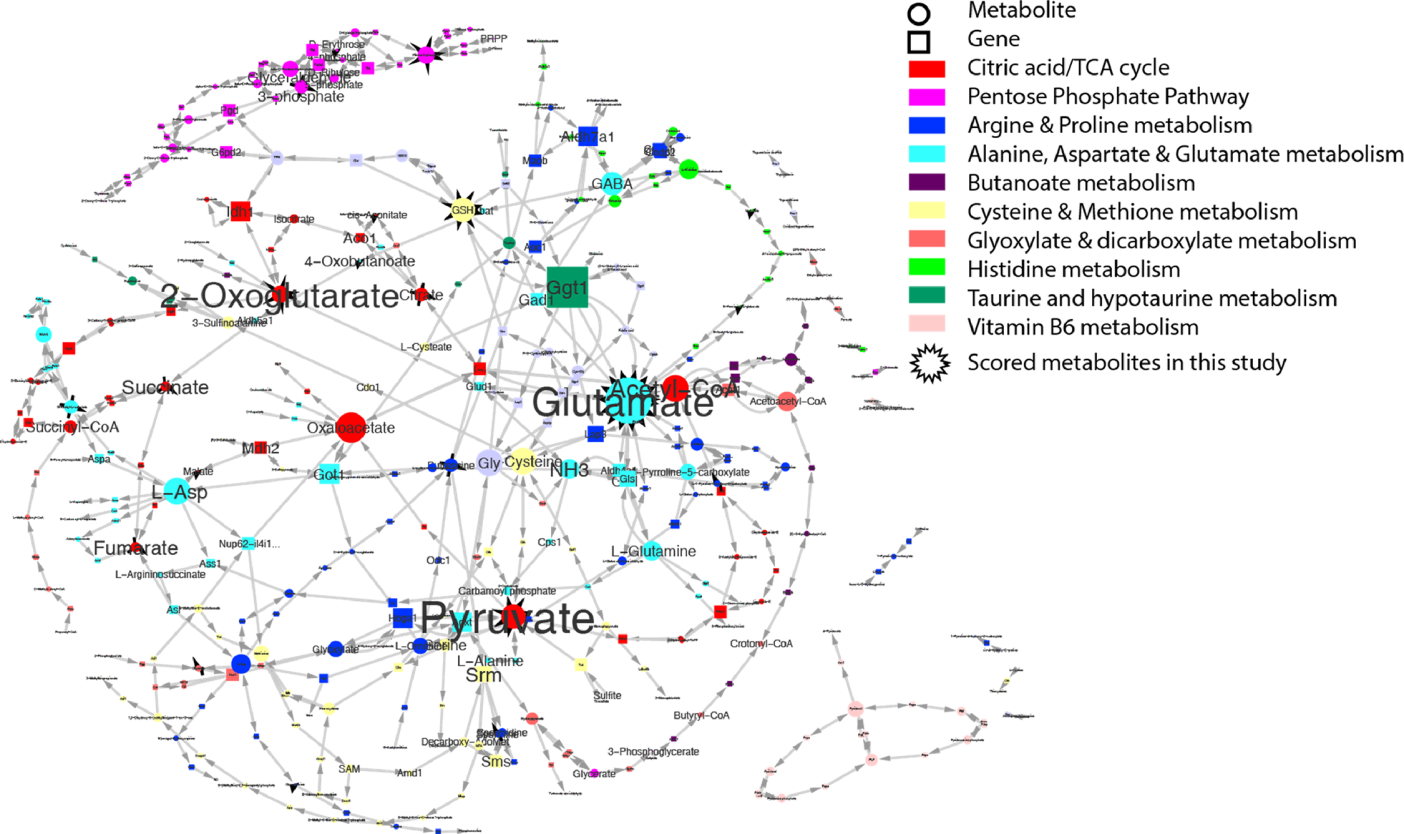
Potential metabolism targets of metformin revealed in this study. KEGG metabolism pathways enriched in this study (Table 1) were merged in a focus network. The font size of each node reflects the connectivity weight (number of edges) in this focus network. Each node represents either a metabolite (circle) or a protein (square). The edges indicate directions of biochemical interactions in this network. Plot was generated in Cytoscape.

To help pinpoint the actual metformin targets, we constructed a focused network by merging all the over-represented mouse metabolic pathways in Table 1 (Q value < 0.01) (Fig 4). The relative importance of individual nodes (metabolites or genes) was assessed by their respective connectivity (the number of edges within the network). Several metabolites emerged as the metabolite hubs for metformin response (N of edges ≥3), including glutamate, pyruvate, oxoglutarate, all of which were highly scored in this study (Fig 3) and was confirmed in BMSCs (S1A Fig). Glutamate acts as an autocrine and/or paracrine signal mediator in osteoblasts and osteoclasts to inhibits bone formation while stimulating bone resorption [33]. The function of glutamate in bone indicates glutamate as a valid metabolic target of metformin since metformin stimulates osteoblasts while suppress osteoclasts. Similarly, metformin significantly reduced the elevated succinate level in MKR bone (Fig 3) and BMSCs (S1B Fig).

Top hub genes include gamma-glutamyltransferase 1 (Ggt1), whose homozygous mutants exhibit abnormal skeletal phenotypes [34], and several others in TCA cycle including Isocitrate dehydrogenase 1(Idh1), MDH2 malate dehydrogenase 2 (Mdh2), and glutamic-oxaloacetic transaminase 1(Got1). These entities demonstrate the range of metformin actions in cells despite that not all of them may be the direct targets of metformin.

## Discussion

As the first-line drug for T2D treatment, metformin has been widely studied and associated with several metabolic processes by clinical and experimental studies, such as enhancing glycolysis by sensitizing glucose uptake and eliminating TCA cycle flux and oxidative phosphorylation [29, 35, 36], antagonizing vitamin B12 [37] and its major metabolic target folate [38], and diverting amino acid metabolism to energy generation [34, 39, 40]. It also appears that the boost in glucose uptake by metformin is independent of insulin [29], and vitamin D (another bone strengthening nutrient) is unaffected by metformin [37]. These processes together depict the complex nature of the cellular outcome from metformin administration which is at least partially due to the basal metabolic status in the context of different tissues/cells.

Here we firstly examined the response of MKR mouse bone to metformin (Fig 1). The advantage of MKR type-2 diabetes model is that its lean phenotype could minimize the interference from other variables like obesity, because fat tissues may substantially impact on bone metabolism and homeostasis [41–43]. Our metabolomic analysis of MKR mouse reveals that metformin targets a wide spectrum of metabolites/pathways. In our study, the elevated levels of TCA cycle metabolites and reduced levels of ATP and NAD in hyperglycemic mice register an abnormal capability of energy regeneration which is common in diabetic conditions. This situation is marked by compensatory mobilization of catabolism from alternative energy sources (e.g. amino acids) in response to energy deficit when supply mismatches demand [44]. However, the metabolic effects accompanying the altered energy generation machinery (e.g. TCA cycle, glycolysis) may not always be desirable, especially when the burden on metabolic waste disposal (e.g. urea cycle) is greatly increased. This may jeopardize the survival of the glucose-starved cells in hyperglycemia and diabetes (such as uric acid, Fig 3) [40]. Metformin is known to indirectly activate AMPK, the starvation rescue switch to increase catabolism [17, 18]. It is therefore intriguing that metformin, a catabolism enhancer, may actually improve hyperglycemic or diabetic conditions where the catabolism is already hyper-active.

Considering that energy sources other than glucose, such as amino acids, are more frequently used in hyperglycemia or diabetes [39], concomitant disposal of nitrogen through urea cycle is under higher pressure to remove excessive nitrogen waste, which often leads to high serum uric acid levels in diabetes [45]. Early observations indicate that metformin interferes with aerobic utilization of metabolic fuels, reducing the utilization of pyruvate and glycerol and decreasing the rate of fat re-esterification [46]. We showed that although metformin elicited similar primary metabolomic changes in WT and MKR mice (Fig 2A), additional suppressions were evident only in the metabolome of MKR mice (Table 2). These metformin-specific metabolites were over-represented not only in energy pathways, but also in nitrogen recycle and amino acid metabolism (Fig 4, Table 1 and Table 2).

Our results support that in addition to activation of catabolism, metformin also enhanced on nitrogen metabolism, such as the arginine/proline metabolism pathway (urea cycle) (Table 1) to energize energy generation and detoxify metabolite waste simultaneously. It also appears that the beneficial effects of metformin on diabetic bone health derive more from detoxification rather than restoring the final energy output to normal levels, as the ATP/NAD levels were unaffected by metformin (Fig 3).

Further metabolomic pathway analysis revealed that metformin specifically suppresses metabolites in non-carbohydrate metabolic pathways such as nitrogen disposal (urea cycle), which could signify a stimulated urea cycle caused by hyperglycemia [28]. The treatment of metformin could ultimately lead to re-established metabolic balance with improved efficiency in diabetic condition.

The fact that glutamate was scored as the top hub metabolic target of metformin in the metabolomic network (Fig 4) is a strongly indicator of the validity of this metabolomic pathway analysis. Glutamate is a fundamental extracellular messenger molecule in many tissues, and is used in bone for both neural and non-neural signaling [47]. High extracellular glutamate inhibits proliferation of osteoblastic cells [48] and preferentially suppresses osteoblastogenesis than adipogenesis through the cystine/glutamate antiporter in mesenchymal stem cells [49]. High glutamate could also stimulate osteoclastogenesis through glutamate-mediated activation of the NF-κB pathway [50]. Diabetes and even early prediabetic insulin resistance are associated with increased levels of glutamate in the circulation [51] which corroborates our observation that glutamate was elevated in MKR bone marrow (Fig 3). Therefore, the ability of metformin to significantly reduce the elevated glutamate levels in MKR mouse bone marrow and BMSCs (S1A Fig) could contribute to metformin’s protective function in diabetic bone. As glutamate is a highly connective metabolite in multiple processes including overall energy balance, redox status, and bone marrow cell differentiation, it is likely that metformin modulates malfunctioning metabolism in diabetic bone marrow that leads to restoration of elevated glutamate. In this regard, our network analysis successfully identified metabolites that have known roles in bone regulation. In addition, other hub metabolites with no known roles in bone could also guide us to identify novel key factors targeted by metformin in regulation of bone homeostasis.

Succinate, another metabolite target revealed by this network, may also regulate bone remodeling through osteoclastogenesis. Metabolically, succinate is well studied as key player in TCA cycle and electron transfer chain. However, to our knowledge, there is no report on the role of either succinate or its receptor in osteoclastogenesis up to date. Current studies on succinate-SUCNR1 signaling were limited to retinal and immune cells [52–56]. Succinate-SUCNR1signaling leads to ERK1/2 activation and NFκB cellular translocation. Interestingly, both phosphorylated ERK1/2 [57–59] and NFκB have a crucial role in osteoclast differentiation and bone resorption [60–63]. A recent in vitro study in HEK293s cells [64] found that SUCNR1 is a Gα(i) coupled receptor that increases intracellular calcium concentrations in an inositol phosphate dependent mechanism via PLCβ activation. Calcium signal in osteoclasts has essential and diverse cellular functions including differentiation and gene transcription [65–67]. These studies indicate that SUCNR1 signaling could elicit the same pathways to regulate osteoclast migration, differentiation and function. In addition, SUCNR1 activation regulates dendritic cell migration and activity in response to succinate [55]. As dendritic cells and osteoclasts are both derived from the hematopoietic cell lineage, succinate activated SUCNR1 signaling could also regulate osteoclast migration, activity and eventually bone resorption. Therefore, it is possible that metformin exerts its anti-bone resorption effects by inhibiting SUCNR1/succinate signaling in bones under diabetic conditions.

## Conclusion

In conclusion, metformin specifically suppresses metabolites in other non-carbohydrate metabolic pathways such as nitrogen disposal (urea cycle), which may suggest improved efficiency that ultimately leads to re-establish the metabolic balance in type 2 diabetic condition. More specifically, glutamate and succinate could be promising mediators for the beneficial effects of metformin in type 2 diabetic bones. Although with limited sample size, our novel finding supports that metformin modulates metabolism in a multiplex manner that alleviates compromised bone metabolism in type 2 diabetics. These findings has to be further evaluated with bigger sample size.

## Supporting Information

S1 Fig Validation of other potential metabolism targets of metformin at cellular level in primary bone marrow stromal cells (BMSCs). Male WT and MKR mice (3-month old) were daily treated with PBS or metformin for 14 days, bone marrow flush out cells from long bones were cultured in MEM Alpha Modification (α-MEM) medium containing L-Glutamine, Ribo- and Deoxyribonucleosides (HyClone, Logan, UT, USA), supplemented with 15% Fetal Bovine Serum (Atlanta Biologicals, GA, USA), 100 μg/mL streptomycin, 100 Units/mL penicillin (Gibco, Grand Island, NY, USA) in a 37°C, 5% (v/v) CO2 and humidified incubator. A week later, the culture with enriched primary BMSCs were harvested for mass spectrum analysis. Each plot shows the mean ± SEM values of respective sample groups for one particular metabolite (n = 4 for WT_PBS, WT_Met, MKR_PBS, n = 3 for MKR_Met, each sample with technical triplicates). The p-values for t-test (two-tailed, unequal variance) are showed whenever significant (< 0.05). Plots were generated in GraphPad Prism 6.
(TIF)
