# Supplementary figures and images for: Metformin Improves Diabetic Bone Health by Re-Balancing Catabolism and Nitrogen Disposal
Source: PLoS One. 2015 Dec 30;10(12):e0146152. doi: 10.1371/journal.pone.0146152 (PMC4696809; doi:10.1371/journal.pone.0146152)

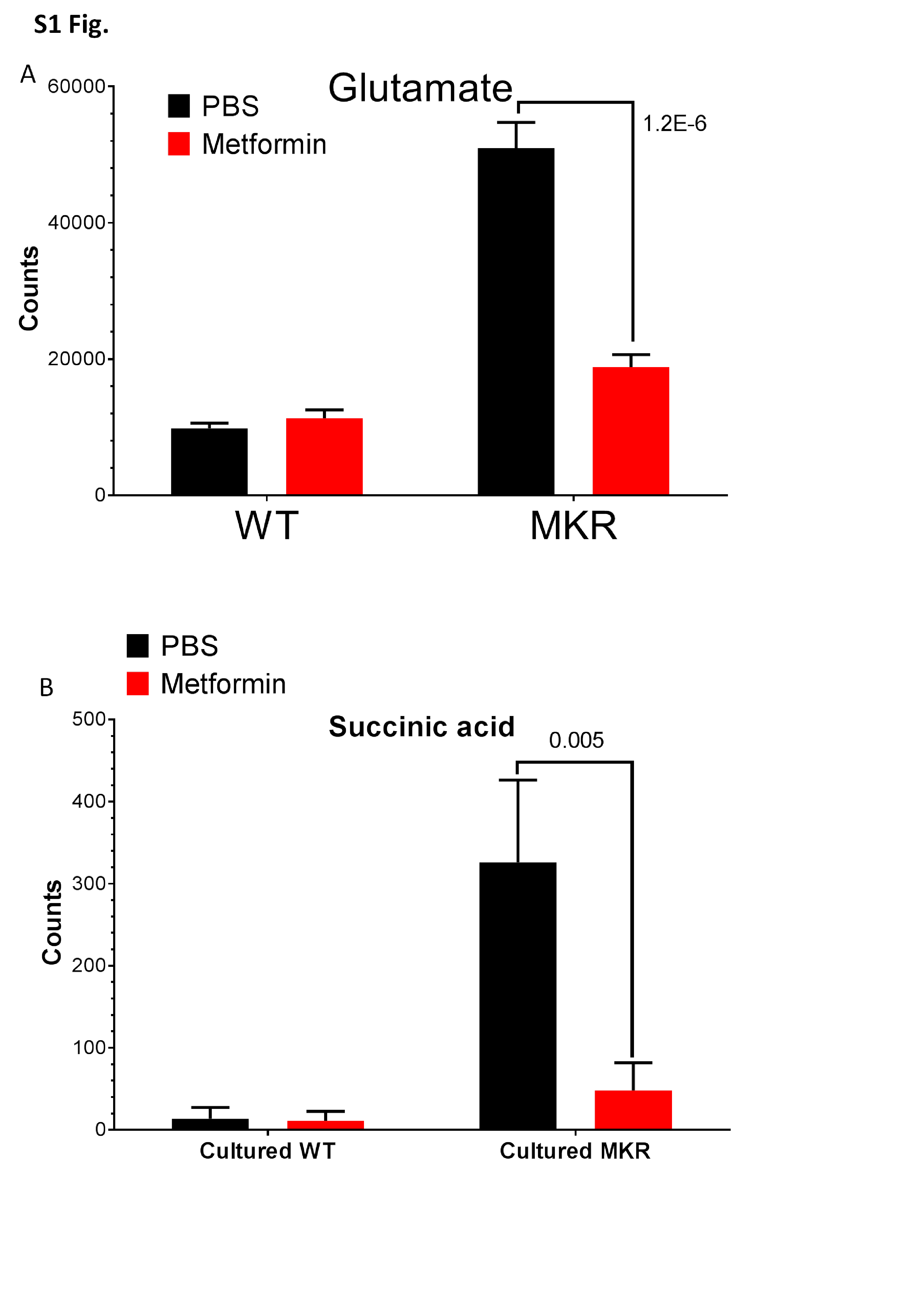

Supplement: S1 Fig — Male WT and MKR mice (3-month old) were daily treated with PBS or metformin for 14 days, bone marrow flush out cells from long bones were cultured in MEM Alpha Modification (α-MEM) medium containing L-Glutamine, Ribo- and Deoxyribonucleosides (HyClone, Logan, UT, USA), supplemented with 15% Fetal Bovine Serum (Atlanta Biologicals, GA, USA), 100 μg/mL streptomycin, 100 Units/mL penicillin (Gibco, Grand Island, NY, USA) in a 37°C, 5% (v/v) CO2 and humidified incubator. A week later, the culture with enriched primary BMSCs were harvested for mass spectrum analysis. Each plot shows the mean ± SEM values of respective sample groups for one particular metabolite (n = 4 for WT_PBS, WT_Met, MKR_PBS, n = 3 for MKR_Met, each sample with technical triplicates). The p-values for t-test (two-tailed, unequal variance) are showed whenever significant (< 0.05). Plots were generated in GraphPad Prism 6. (TIF) [file pone.0146152.s001.tif]
